# Draft Genome Sequence of the Type Strain Bacillus subtilis subsp. *subtilis* DSM10

**DOI:** 10.1128/MRA.00158-21

**Published:** 2021-03-11

**Authors:** Lars Lilge, Robert Hertel, Kambiz Morabbi Heravi, Marius Henkel, Fabian M. Commichau, Rudolf Hausmann

**Affiliations:** a University of Hohenheim, Institute of Food Science and Biotechnology, Department of Bioprocess Engineering (150k), Stuttgart, Germany; b Brandenburg University of Technology, FG Synthetic Microbiology, Cottbus-Senftenberg, Germany; Indiana University, Bloomington

## Abstract

The Bacillus subtilis subsp. *subtilis* type strain DSM10 has been used as a reference in various studies. However, detailed information about the genome has not been available. Therefore, whole-genome sequencing was performed, and the sequence was compared with that of the related B. subtilis strain NCIB3610.

## ANNOUNCEMENT

The Bacillus subtilis subsp. *subtilis* type strain DSM10 is a generally accessible *Bacillus* strain from the German Collection of Microorganisms and Cultures GmbH (DSMZ). It has been used as a reference strain for applied biotechnological research (1–4). For instance, the DSM10^T^ strain produces notable amounts of surfactin (2, 5, 6) and secretes proteases (data not shown). Due to its descent from the B. subtilis Marburg strain (7), it is feasible to genetically engineer the DSM10^T^ strain, making it a promising bacterial system for basic research and industrial strain engineering. According to the DSMZ, B. subtilis DSM10^T^ corresponds to B. subtilis strain NCIB3610 (8). Whole-genome sequencing of B. subtilis DSM10^T^ was performed to verify this assumption.

A single colony was inoculated in LB medium and cultivated overnight at 37°C and 120 rpm. Subsequently, chromosomal DNA was extracted with an innuPrep bacterial DNA kit (Analytik Jena, Jena, Germany). Library preparation and whole-genome sequencing were performed by Eurofins Genomics (Ebersberg, Germany). An Illumina HiSeq 4000 system was employed for sequencing, resulting in 2 × 101-bp paired-end read files. The paired-end reads obtained (2 × 35.9 million) were quality analyzed with FastQC v0.11.9 (9). A subset of 2 × 5 million reads, randomly extracted with seqtk v1.3-r106 (10), were used for short-read assembly with SPAdes v3.14.0 (11) with the option “careful.” This resulted in 26 contigs of >0.2 kb with a total size of 4,166,758 bp, an *N*_50_ value of 1,014,761 bp, and an *N*_90_ value of 240,612 bp. The genomes of the sibling strain B. subtilis NCIB3610 (GenBank accession number CP020102) and its plasmid pBS32 (GenBank accession number CP020103) were used as references to sort and correctly orient the acquired draft genome of B. subtilis DSM10^T^ with the program Mauve v2015-02-13 (http://www.darlinglab.org/mauve). The alignment obtained revealed an almost perfect match of the DSM10^T^ draft genome to the chromosome of NCIB3610. However, contigs resembling pBS32 were not observed, confirming its absence in DSM10^T^. The DSM10^T^ genome underwent automated gene annotation by the Prokaryotic Genome Annotation Pipeline (PGAP) during uploading to GenBank (12). This process led to the identification of 4,289 genes, of which 4,252 are protein-coding genes and 37 are pseudogenes. Moreover, 42 tRNAs and 5 noncoding RNAs were identified and annotated. The program breseq v0.35.1 (13) was used to identify specific differences by applying DSM10 reads to the NCIB3610 genome. In all, 39 sequence variations could be identified (Table 1). Seventeen single-nucleotide polymorphisms restored a corresponding NCIB3610 pseudogene.

**TABLE 1:** Sequence variations between B. subtilis DSM10^T^ and NCIB3610

Contig accession no.	Nucleotide position	Mutation	Annotation^*a*^	Gene	Description^*b*^
JAEPVU010000002.1	26036	C→T	P307L (CCG→CTG)	*mfd* →	Transcription repair coupling factor
JAEPVU010000003.1	11953	−G	Gene-pseudogene	*cysE* →	Serine *O*-acetyltransferase
JAEPVU010000006.1	105978	(ATGATAGT)1→2	Intergenic	H9S96_03325 ← / → H9S96_03330	Catalase/tRNA-Arg
JAEPVU010000007.1	83281	(G)7→6	Gene-pseudogene	*yerH* →	Hypothetical protein
JAEPVU010000007.1	85980	(C)6→5	Gene-pseudogene	*sapB* ←	Methyltransferase
JAEPVU010000007.1	141088	T→C	V142A (GCG→GTG)	*lplB* →	Protein lplB
JAEPVU010000007.1	147278	(G)6→5	Intergenic	H9S96_04045 → / → *yezD*	Sulfate transporter/DUF2292 domain-containing protein
JAEPVU010000007.1	241945	(G)6→5	Gene-pseudogene	*acoL* →	Dihydrolipoyl dehydrogenase
JAEPVU010000009.1	94306	(A)7→6	Gene-pseudogene	*bmrD (yheH)* →	Multidrug ABC transporter permease
JAEPVU010000009.1	234838	−ATGTAC	Coding	*yitS* ←	Fatty acid-binding protein DegV
JAEPVU010000009.1	320518	(G)6→5	Gene-pseudogene	*manP* →	PTS mannose transporter subunit IIABC
JAEPVU010000009.1	357081	−G	Gene-pseudogene	*uxaB* →	Altronate oxidoreductase
JAEPVU010000009.1	358313	(G)6→5	Gene-pseudogene	*uxaB* →	Altronate oxidoreductase
JAEPVU010000009.1	375395	(C)6→5	Gene-pseudogene	*xkdE* →	Phage portal protein
JAEPVU010000009.1	471575	(TAAT)4→3	Intergenic	*mtnK* ← / ← *mtnU*	*S*-Methyl-5-thioribose kinase/hydrolase MtnU
JAEPVU010000009.1	514727	(T)8→7	Intergenic	*ykwD* ← / → *pbpH*	Hypothetical protein/penicillin-binding protein
JAEPVU010000009.1	737764	A→G	I176V (ATC→GTC)	*codY* →	GTP-sensing pleiotropic transcriptional regulator CodY
JAEPVU010000009.1	956741	A→G	S179G (AGT→GGT)	*yndE* →	Germination protein
JAEPVU010000011.1	175058	−C	Coding (131/138 nt)	*yotE* ←	Hypothetical protein
JAEPVU010000011.1	222781	C→A	V91V (GTG→GTT)	*yopW* ←	Hypothetical protein
JAEPVU010000011.1	379673	(C)6→5	Intergenic	*mgsA* ← / ← *dapB*	Methylglyoxal synthase/4-hydroxy-tetrahydrodipicolinate reductase
JAEPVU010000011.1	396124	(C)7→6	Gene-pseudogene	*trpD* ←	Anthranilate phosphoribosyltransferase
JAEPVU010000011.1	534030	(C)7→6	Gene-pseudogene	*mmgA* ←	Acetyl-CoA acetyltransferase
JAEPVU010000011.1	638280	−C	Gene-pseudogene	*yqfA* (*floA*)←	Hypothetical protein
JAEPVU010000011.1	719953	(T)8→7	Gene-pseudogene	*yqaD* ←	Hypothetical protein
JAEPVU010000011.1	728398	(C)8→7	Intergenic	*yrkN* → / ← *yrkL*	*N*-Acetyltransferase/general stress protein
JAEPVU010000011.1	749018	(C)6→5	Gene-pseudogene	*azlD* ←	Branched-chain amino acid transporter AzlD
JAEPVU010000011.1	752943	(C)6→5	Intergenic	*cypA* ← / ← *yrdC*	Cytochrome P450/cysteine hydrolase
JAEPVU010000011.1	875960	A→G	G66G (GGT→GGC)	*rplU* ←	50S ribosomal protein L21
JAEPVU010000011.1	900227	(T)7→6	Coding	*engB* ←	YihA family ribosome biogenesis GTP-binding protein
JAEPVU010000011.1	982769	(C)6→5	Intergenic	*ytxC* ← / ← *ytxB*	Hypothetical protein/TVP38/TMEM64 family membrane protein YtxB
JAEPVU010000011.1	1079814	(C)8→7	Intergenic	*ytnP* ← / ← *trmB*	MBL fold metallo-hydrolase/tRNA (guanosine-46-N7)-methyltransferase TrmB
JAEPVU010000012.1	88	A→G	Noncoding	H9S96_16680 ←	tRNA-Ala
JAEPVU010000013.1	61274	(G)6→5	Intergenic	*malR* (*yufM*) → / → *yufN* (*nupN*)	Two-component system response regulator DcuR/BMP family ABC transporter substrate-binding protein
JAEPVU010000013.1	227225	(T)6→5	Gene-pseudogene	*yvrE* ←	Sugar lactone lactonase YvrE
JAEPVU010000013.1	317921	−C	Gene-pseudogene	*yvfT* ←	Two-component sensor histidine kinase
JAEPVU010000013.1	401206	(T)8→7	Gene-pseudogene	*yvzA* ←	Hypothetical protein
JAEPVU010000013.1	414816	G→A	L256F (CTC→TTC)	*lgt* ←	Prolipoprotein diacylglyceryl transferase
JAEPVU010000014.1	21905	G→T	F118L (TTC→TTA)	*rocB* ←	Peptidase M20
JAEPVU010000014.1	220111	A→G	L25L (TTG→CTG)	*iolG* ←	Inositol 2-dehydrogenase

a Variations identified between B. subtilis strain DSM10^T^ and strain NCIB3610. nt, nucleotides.

b PTS, phosphotransferase system; CoA, coenzyme A.

In conclusion, B. subtilis DSM10^T^ is genomically very similar to NCIB3610 but is not identical. The absence of pBS32 could explain the ability of DSM10^T^ to develop competence because no plasmid-borne single-pass transmembrane protein ComI is present to downregulate it, as it is for NCIB3610 (14).

### Data availability.

The genome sequence of B. subtilis subsp. *subtilis* strain DSM10^T^ has been deposited in GenBank under the accession number JAEPVU000000000. The raw sequence reads have been submitted to the NCBI Sequence Read Archive (SRA) (15) under the accession number SRR12632401. The BioProject accession number is PRJNA659394, and the BioSample accession number is SAMN15904628.
